# Coronary angiography is related to improved clinical outcome of out-of-hospital cardiac arrest with initial non-shockable rhythm

**DOI:** 10.1371/journal.pone.0189442

**Published:** 2017-12-29

**Authors:** Eunsil Ko, Ji Kyoung Shin, Won Chul Cha, Joo Hyun Park, Tae Rim Lee, Hee Yoon, Guntak Lee, Sung Yeon Hwang, Tae Gun Shin, Min Seob Sim, Ik Joon Jo, Joong Eui Rhee, Keun Jeong Song, Yeon Kwon Jeong, Sang Do Shin, Jin-Ho Choi, Cardiac Arrest Pursuit Trial with Unique Registry, Epidemiologic Surveillance investigators

**Affiliations:** 1 Department of Emergency Medicine, Samsung Medical Center, Sungkyunkwan University School of Medicine, Seoul, Republic of Korea; 2 Department of Emergency Medicine, Seoul National University College of Medicine, Seoul, Republic of Korea; Osaka University Graduate School of Medicine, JAPAN

## Abstract

**Objective:**

Coronary angiography (CAG) for survivors of out-of-hospital cardiac arrest (OHCA) enables early identification of coronary artery disease and revascularization, which might improve clinical outcome. However, little is known for the role of CAG in patients with initial non-shockable cardiac rhythm.

**Methods:**

We investigated clinical outcomes of successfully resuscitated 670 adult OHCA patients who were transferred to 27 hospitals in Cardiac Arrest Pursuit Trial with Unique Registration and Epidemiologic Surveillance (CAPTURES), a Korean nationwide multicenter registry. The primary outcome was 30-day survival with good neurological outcome. Propensity score matching and inverse probability of treatment weighting analyses were performed to account for indication bias.

**Results:**

A total of 401 (60%) patients showed initial non-shockable rhythm. CAG was performed only in 13% of patients with non-shockable rhythm (53 out of 401 patients), whereas more than half of patients with shockable rhythm (149 out of 269 patients, 55%). Clinical outcome of patients who underwent CAG was superior to patients without CAG in both non-shockable (hazard ratio (HR) = 3.6, 95% confidence interval (CI) = 2.5–5.2) and shockable rhythm (HR = 3.7, 95% CI = 2.5–5.4, *p* < 0.001, all). Further analysis after propensity score matching or inverse probability of treatment weighting showed consistent findings (HR ranged from 2.0 to 3.2, *p* < 0.001, all).

**Conclusions:**

Performing CAG was related to better survival with good neurological outcome of OHCA patients with initial non-shockable rhythms as well as shockable rhythms.

## Introduction

Out-of-hospital cardiac arrest (OHCA) is a major public health burden and associated with high morbidity and mortality rates worldwide [1]. Coronary artery disease is the most common cause of adult OHCA [2, 3]. Therefore, improved survival among OHCA survivor who underwent coronary angiography (CAG) can be easily anticipated because CAG enables immediate diagnosis and appropriate treatment including revascularization [4, 5]. The clinical benefit of CAG has been studied mostly for adult OHCA patients with initial shockable rhythm such as ventricular tachyarrhythmia [4, 5]. However, it is not well known whether performing CAG has clinical benefit in OHCA patient with initial non-shockable rhythms including pulseless electrical activity (PEA) and asystole.

Most improvement in the clinical outcome of OHCA has been derived from improved survival in OHCA with initial shockable rhythm [6, 7], whereas OHCA with initial non-shockable rhythm still suffers from little improvement of very poor clinical outcome [8–11]. In addition, the proportion of initial non-shockable rhythm to the initial shockable rhythm has increased consistently [12–14]. The aim of this study was to assess the association between CAG and clinical outcomes in adult non-shockable OHCA from Korean nationwide multicenter OHCA registry. Propensity score matching and inverse probability of treatment weighting analyses were applied to minimize indication bias.

## Methods

### Data source and study population

**Fig 1:**
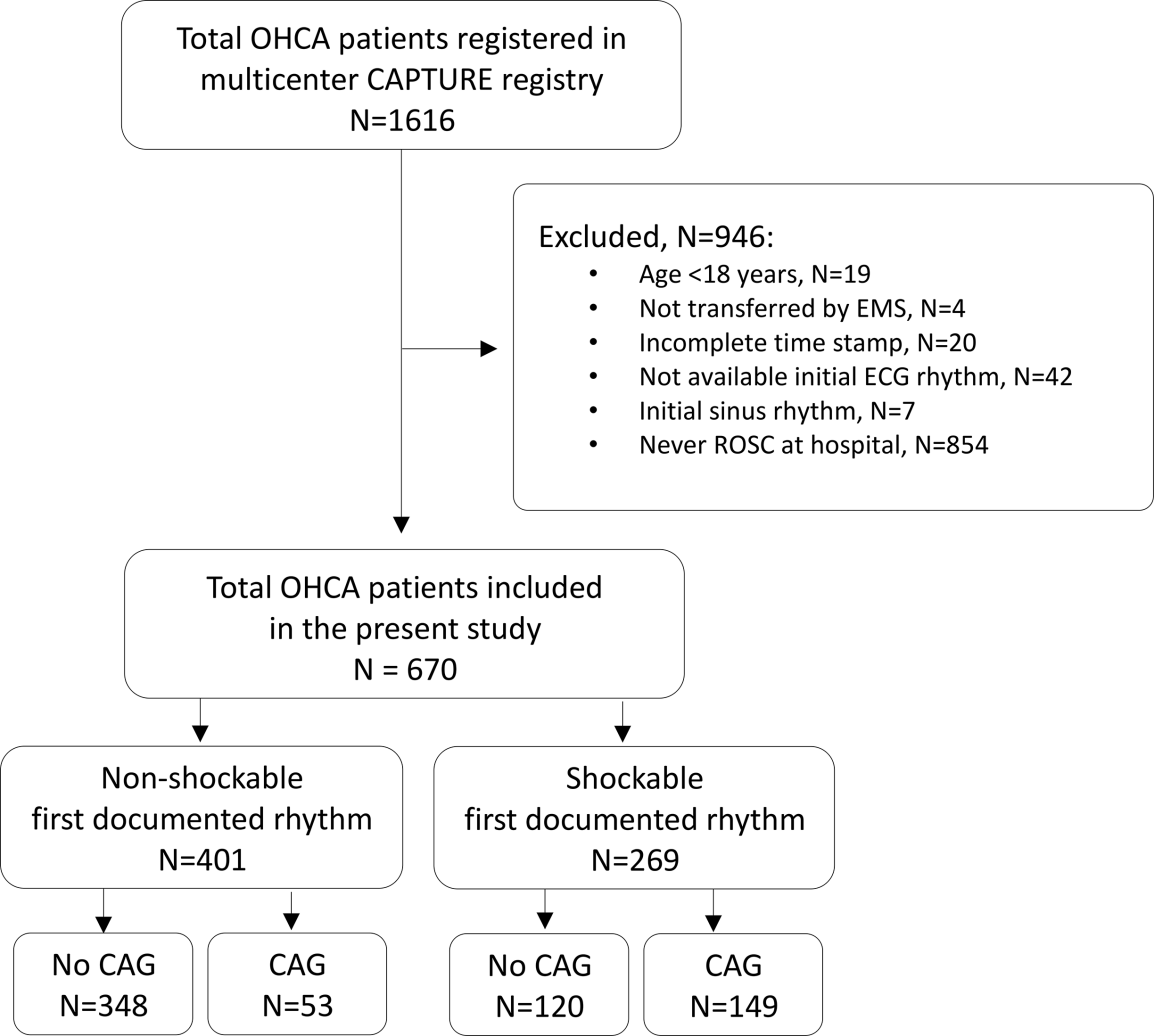
Study population flow. Abbreviations: OHCA, out-of-hospital cardiac arrest; CAPTURES, Cardiac Arrest Pursuit Trial with Unique Registration and Epidemiologic Surveillance; EMS, emergency medical service; ECG, electrocardiography; ROSC, return of spontaneous circulation; CAG, coronary angiography.

The Cardiac Arrest Pursuit Trial with Unique Registration and Epidemiologic Surveillance (CAPTURES) project was a Korean multicenter observational study conducted from January to December 2014 at 27 emergency departments (EDs) (9 level 1 EDs and 18 level 2 EDs). This project aimed to identify the risk factors of OHCA and to evaluate the prognostic factors in long-term follow up. The CAPTURES project included OHCA patients who were transported to the study EDs with resuscitation efforts and had a presumed cardiac etiology as identified by emergency physicians in each ED. OHCA patients with definite non-cardiac etiology such as trauma, drowning, hanging, poisoning, asphyxia, burn, hemorrhagic or ischemic stroke, or terminal illness were excluded. Clinical data were collected at each study ED and transferred to the central data server using EpiData version 3.1 (The EpiData Association, Denmark, Europe). A total of 1616 OHCA patients were registered in CAPTURES project. After exclusion of patients who could not achieve a return of spontaneous circulation (ROSC, N = 854), were not transferred by emergency medical service or lacks documentation (N = 66), age < 18 (N = 19), and initial sinus rhythm (N = 7), 670 OHCA patients consisting of 401 initial non-shockable rhythm and 269 initial shockable rhythm were enrolled in the analysis (Fig 1).

### Clinical variables

Pre-hospital patient-level data included age, gender, location of arrest (public, home or healthcare), bystander witness, bystander cardiopulmonary resuscitation (CPR), prehospital defibrillation, ED arrival at weekend or business hour, and first documented cardiac rhythm dichotomized as non-shockable or shockable rhythm. We also calculated time interval of response time (from EMS call time to EMS arrival time) and transfer time (from EMS arrival time to ED arrival time) based on timestamp of the medical record.

Cardiovascular risk factors including diabetes mellitus, hypertension and dyslipidemia were assessed if available. In-hospital data included intubated status, use of intravenous inotropic agent, CAG, revascularization by percutaneous coronary revascularization (PCI), mechanical circulatory support including intra-aortic balloon pump (IABP) or extracorporeal membrane oxygenation (ECMO), implantation of implantable cardioverter defibrillator (ICD) and targeted temperature management (TTM).

### Study outcome

The primary outcome was survival with a favorable neurological outcome at discharge measured by the Glasgow-Pittsburgh cerebral performance category (CPC) scores at 30 days. Good and poor neurological outcome was defined by CPC = 1 or 2 and CPC = 3 to 5, respectively. The secondary outcome was all-cause death within 30 days.

### Statistical analysis

**Fig 2:**
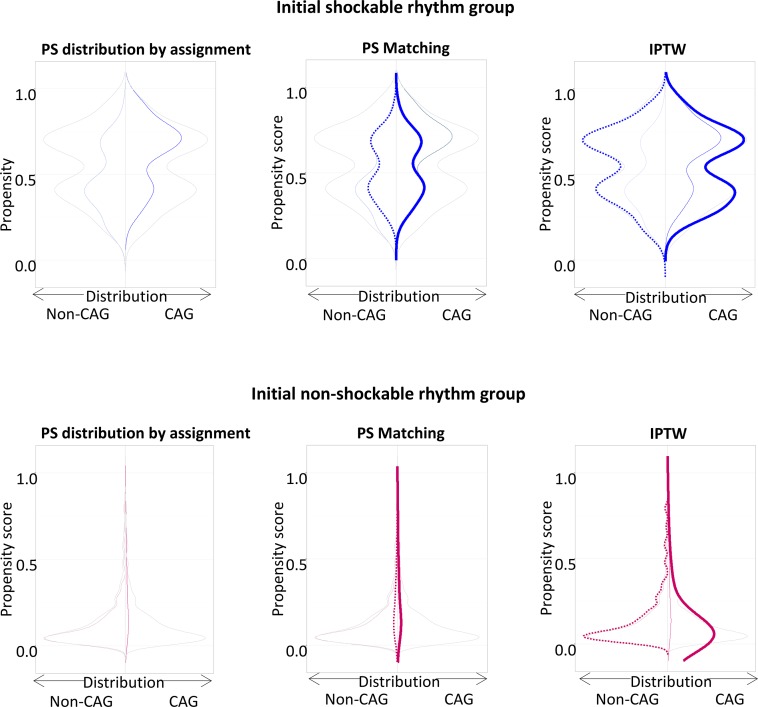
PS matching and IPTW analyses for adult OHCA patients according to initial ECG rhythm. Abbreviations: PS, propensity score; IPTW, inverse probability of treatment weighting; OHCA, out-of-hospital cardiac arrest; ECG, electrocardiography; CAG, coronary angiography.

Continuous and categorical data are presented as median with 1^st^–3^rd^ quartile and number (proportion, %), respectively, and tested by Mann Whitney U or chi-square test, appropriately. The Cox proportional hazards model was used to estimate the hazard ratio (HR) and 95% confidence interval (CI) for clinical outcomes between groups. Comparison was adjusted by propensity score matching (PSM) and inverse probability of treatment weighting (IPTW) to reduce indication bias. For PSM analysis, 1:1 matched pairs were selected based on the predicted probability of being assigned to CAG in non-shockable and shockable OHCA. Non-parsimonious model was developed including clinical characteristics variables; age, gender, location of arrest, bystander witness and resuscitation, prehospital defibrillation, ED visit at business hour, intubated status, use of intravenous inotropic agent, and TTM. Discrimination and calibration performance of the model was tested by c-statistics (0.794 for non-shockable rhythm group, 0.733 for shockable rhythm group) and Hosmer-Lemeshow statistics (chi-square = 9.38, df = 8, *p* = 0.31 for non-shockable rhythm group, chi-square = 2.04, df = 8, *p* = 0.98 for shockable rhythm group), respectively. The distributions of propensity score in PSM and IPTW analyses were shown in Fig 2. Statistical significance was defined by two-tailed p value < 0.001. R version 3.4 (R foundation for Statistical Computing, Vienna, Austria) was used for all computational analyses.

### Ethics statements

The study protocol was approved by all Institutional Review Boards of 27 participating hospitals with waiver of informed consent. This study was financially supported by the Korea Centers for Disease Control and Prevention (2013–2014).

## Results

### Clinical characteristics

Compared to patients with shockable rhythm, patients with non-shockable rhythm were older (median age 67 versus 56 years), more likely to be female (34 versus 19%), and showed higher frequency of clinical risk factors (*p* < 0.001, all). Patients with non-shockable rhythm were less likely to be found at public location (23 versus 47%), be witnessed (68 versus 81%), and receive bystander CPR (39 versus 60%, *p* < 0.001, all). Time from EMS call to EMS arrival was not different between two groups but time from EMS arrival to ED transfer was shorter in non-shockable rhythm group compared to shockable rhythm (19 versus 24 minutes). Patients with non-shockable rhythm underwent more intubation (96 versus 87%), use of inotropics (82 versus 62%), but less CAG (13 versus 55%) and TTM (22 versus 45%, *p* < 0.001, all) (Table 1).

**Table 1:** Baseline characteristics of adult OHCA patients according to initial ECG rhythm.

	Non-shockable rhythm (N = 401)	Shockable rhythm (N = 269)	*p*-value
**Age, median [IQR]**	67 [53, 77]	56 [47, 67]	<0.001
**Female, N(%)**	136 (34)	52 (19)	<0.001
**Hypertension, N(%)**	169 (42)	95 (35)	0.009
**DM, N(%)**	98 (24)	46 (17)	0.005
**Dyslipidemia, N(%)**	21 (5)	20 (7)	0.077
**Arrest location, N(%)**			<0.001
** Home**	249 (62)	111 (41)	
**Public**	94 (23)	127 (47)	
**Healthcare**	50 (13)	20 (7)	
**Unknown**	8 (2)	11 (4)	
**Bystander witnessed, N(%)**	272 (68)	217 (81)	<0.001
**Bystander CPR, N(%)**	156 (39)	161 (60)	<0.001
**First documented rhythm, N(%)**			
**Asystole**	251 (63)		
**PEA**	110 (27)		
**Unknown**	40 (10)		
**Prehospital defibrillation, N(%)**	33 (8)	251 (93)	<0.001
Min to response*, median [IQR]	6.0 [5.0, 9.0]	6.0 [5.0, 8.0]	0.493
Min to transfer*, median [IQR]	19.0 [13.0, 28.0]	24.0 [15.0, 59.0]	<0.001
**ED visit at working hour** **(8 a.m. to 5 p.m.), N(%)**	111 (28)	68 (25)	0.549
**ED visit at weekend, N(%)**	100 (25)	100 (37)	0.001
**Hospital Course**			
**Intubation, N(%)**	383 (96)	235 (87)	<0.001
**Use of inotropics, N(%)**	329 (82)	167 (62)	<0.001
**CAG, N(%)**	53 (13)	149 (55)	<0.001
**PCI, N(%)**	20 (5)	54 (20)	<0.001
**IABP, N(%)**	4 (1)	18 (7)	<0.001
**ECMO, N(%)**	12 (3)	20 (7)	0.014
**Implantation of ICD, N(%)**	2 (1)	29 (11)	<0.001
**Temporary pacing, N(%)**	4 (1)	7 (3)	0.196
**TTM, N(%)**	89 (22)	120 (45)	<0.001

Abbreviations: OHCA, out-of-hospital cardiac arrest; ECG, electrocardiography; IQR, interquartile range; DM, diabetes mellitus; CPR, cardiopulmonary resuscitation; PEA, pulseless electrical activity; ED, emergency department; CAG, coronary angiography; PCI, percutaneous coronary intervention; IABP, intra-aortic balloon pump; ECMO, extracorporeal membrane oxygenation; ICD, implantable cardioverter defibrillator; TTM, targeted temperature management

*Min to response, minutes from EMS call to EMS arrival; Min to transfer, minutes from EMS arrival to ED arrival.

When these clinical characteristics were classified by CAG, patients with non-shockable rhythm who underwent CAG were more likely to be found at public location (45 versus 20%), receive prehospital defibrillation (26% versus 6%), and TTM (43 versus 19%, *p* < 0.001, all). Patients with shockable rhythm who underwent CAG were less likely to receive an advanced airway (81 versus 96%) and more likely to receive TTM (55 versus 32%, *p* < 0.001, all). Among patients who underwent CAG, the frequency of ST-elevation ECG (21% versus 18%, *p* = 0.68) and the frequency of revascularization by PCI (38% versus 36%, *p* = 0.28) was not different between non-shockable and shockable rhythm (Table 2).

**Table 2:** Baseline characteristics of adult OHCA patients according to initial ECG rhythm and CAG.

	Non-shockable rhythm			Shockable rhythm		
	Non-CAG N = 348	CAG N = 53	*p*-value	Non-CAG N = 120	CAG N = 149	*p*-value
**Age, median [IQR]**	69 [54, 79]	57 [53, 69]	0.001	58 [45, 70]	54 [48, 65]	0.444
**Female, N(%)**	128 (37)	8 (15)	0.003	32 (27)	20 (13)	0.01
**Hypertension, N(%)**	147 (42)	22 (42)	0.83	35 (29)	60 (40)	0.112
**DM, N(%)**	85 (24)	13 (25)	0.872	22 (18)	24 (16)	0.295
**Dyslipidemia, N(%)**	18 (5)	3 (6)	0.83	6 (5)	14 (9)	0.257
**Arrest location, N(%)**			<0.001			0.348
**Home**	231 (66)	18 (34)		50 (42)	61 (41)	
**Public**	70 (20)	24 (45)		59 (49)	68 (46)	
**Healthcare**	42 (12)	8 (15)		9 (8)	11 (7)	
**Unknown**	5 (1)	3 (6)		2 (2)	9 (6)	
**Bystander witnessed, N(%)**	229 (66)	43 (81)	0.039	100 (83)	117 (79)	0.402
**Bystander CPR, N(%)**	133 (38)	23 (43)	0.569	63 (53)	98 (66)	0.037
**First documented rhythm, N(%)**			<0.001			
**Asystole**	230 (66)	21 (40)				
**PEA**	89 (26)	21 (40)				
**Unknown**	29 (8)	11 (21)				
**ST-elevation in post-ROSC ECG**	17 (5)	11 (21)	<0.001	5 (4)	27 (18)	<0.001
**Prehospital defibrillation, N(%)**	19 (6)	14 (26)	<0.001	110 (92)	141 (95)	0.47
Min to response*, median [IQR]	6.0 [5.0, 8.0]	7.0 [5.0, 10.0]	0.274	6.0 [5.0, 8.0]	6.0 [5.0, 8.0]	0.735
Min to transfer*, median [IQR]	19.0 [14.0, 27.0]	18.0 [13.0, 37.0]	0.828	23.0 [15.0, 55.3]	24.0 [15.0, 71.0]	0.6
**ED visit at working hour** **(8 a.m. to 5 p.m.), N(%)**	101 (29)	10 (19)	0.169	31 (26)	37 (25)	0.963
**ED visit at weekend, N(%)**	89 (26)	11 (21)	0.558	45 (38)	55 (37)	1
**Hospital Course**						
**Intubation, N(%)**	331 (95)	52 (98)	0.531	115 (96)	120 (81)	<0.001
**Use of inotropics, N(%)**	285 (82)	44 (83)	0.995	77 (64)	90 (60)	0.613
**PCI, N(%)**	0 (0)	20 (38)	<0.001	0 (0)	54 (36)	<0.001
**IABP, N(%)**	1 (0.3)	3 (6)	0.003	0 (0)	18 (12)	<0.001
**ECMO, N(%)**	6 (2)	6 (11)	0.001	2 (2)	18 (12)	0.003
**Implantation of ICD, N(%)**	0 (0)	2 (4)	0.01	8 (7)	21 (14)	0.079
**Temporary pacing, N(%)**	2 (0.6)	2 (4)	0.15	3 (3)	4 (3)	1
**TTM, N(%)**	66 (19)	23 (43)	<0.001	38 (32)	82 (55)	<0.001

Abbreviations: OHCA, out-of-hospital cardiac arrest; ECG, electrocardiography; CAG, coronary angiography; IQR, interquartile range; DM, diabetes mellitus; CPR, cardiopulmonary resuscitation; PEA, pulseless electrical activity; ED, emergency department; PCI, percutaneous coronary intervention; IABP, intra-aortic balloon pump; ECMO, extracorporeal membrane oxygenation; ICD, implantable cardioverter defibrillator; TTM, targeted temperature management

*Min to response, minutes from EMS call time to EMS arrival time; Min to transfer, minutes from EMS arrival time to ED arrival time.

### Comparisons of clinical outcomes according to initial electrocardiogram rhythm and CAG

**Fig 3:**
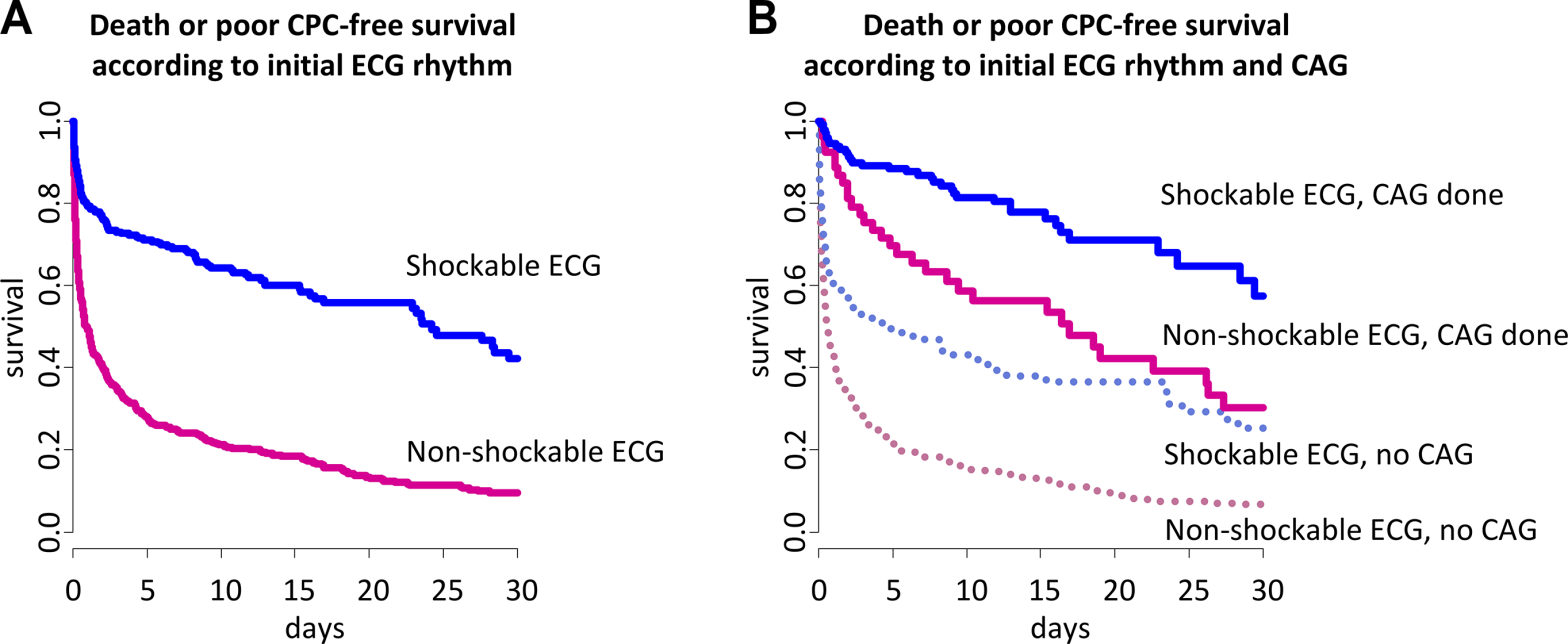
Unadjusted survival curves for adult OHCA patients. (A) Unadjusted survival curves according to initial ECG rhythm. (B) Unadjusted survival curves according to CAG. Abbreviations: OHCA, out-of-hospital cardiac arrest; ECG, electrocardiography; CAG, coronary angiography; CPC, cerebral performance category.

Patients with shockable rhythm showed much higher 30-day survival with good neurological outcome compared with patients with non-shockable rhythm (unadjusted HR = 3.2, 95% CI = 2.6–3.9, *p* < 0.001) (Fig 3A). Intriguingly, performing CAG is associated with better clinical outcome in both non-shockable and shockable rhythm group (Fig 3B). As shown in Table 3, the unadjusted 30-day survival with good neurological outcome was more than 3-fold higher in patients underwent CAG compared with patients without CAG irrespective of initial rhythm (non-shockable rhythm group, HR = 3.6 (95% CI = 2.5–5.2); shockable rhythm group, HR = 3.7 (95% CI = 2.5–5.4), respectively, *p* < 0.001, all). After PSM or IPTW adjustment, the 30-day survival with good neurological outcome was still more than 2-fold higher in patients underwent CAG compared with patients without CAG irrespective of initial rhythm (PSM, non-shockable rhythm group, HR = 2.4 (95% CI = 1.5–3.8); shockable rhythm group, HR = 2.3 (95% CI = 1.5–3.7), respectively, *p* < 0.001, all; IPTW, non-shockable rhythm group, HR = 2.0 (95% CI = 1.5–2.7); shockable rhythm group, HR = 3.2 (95% CI = 2.2–4.7), respectively, *p* < 0.001, all). The superior clinical outcome of patients with CAG was also maintained when analyzed with the 30-day all-cause death (Table 3).

**Table 3:** Unadjusted and adjusted hazard ratios for adult OHCA patients with initial non-shockable or shockable rhythm.

**Initial shockable rhythm**								
	**Clinical outcome**		**Crude (N = 269)**		**PS match (85 pairs)**		**IPTW**	
	Non-CAG (N = 120)	CAG (N = 149)	HR (95% CI)	*p*-value	HR (95% CI)	*p*-value	HR (95% CI)	*p*-value
**Survival with good CPC at 30 day**	35 (29)	110 (74)	3.7 (2.5–5.4)	<0.001	2.3 (1.5–3.7)	<0.001	3.2 (2.2–4.7)	<0.001
**Survival at 30 day**	49 (41)	121 (81)	4.4 (2.8–6.8)	<0.001	2.6 (1.5–4.3)	<0.001	3.5 (2.3–5.5)	<0.001
**Initial non-shockable rhythm**								
	**Clinical outcome**		**Crude (N = 401)**		**PS match (46 pairs)**		**IPTW**	
	Non-CAG (N = 348)	CAG (N = 53)	HR (95% CI)	*p*-value	HR (95% CI)	*p*-value	HR (95% CI)	*p*-value
**Survival with good CPC at 30 day**	14 (4)	19 (36)	3.6 (2.5–5.2)	<0.001	2.4 (1.5–3.8)	<0.001	2.0 (1.5–2.7)	<0.001
**Survival at 30 day**	61 (18)	30 (57)	3.8 (2.5–5.8)	<0.001	2.5 (1.4–4.3)	0.001	2.1 (1.5–3.0)	<0.001

Abbreviations: OHCA, out-of-hospital cardiac arrest; PS, propensity score; IPTW, inverse probability of treatment weighting; CAG, coronary angiography; HR, hazard ratio; CI, confidence interval; CPC, cerebral performance category.

## Discussion

In this study based on a nationwide multicenter OHCA registry, we found that patients who underwent CAG after successful ROSC had better short-term survival with favorable neurological status than those who did not. The better clinical outcome of performing CAG was found not only in shockable OHCA but also in non-shockable OHCA. The result was also consistent after PSM and IPTW adjusted analyses.

Prior observational studies have reported that CAG was associated with improved clinical outcomes in OHCA survivors [5, 15–18]. Most previous studies have mainly included OHCA patients with initial shockable rhythm because acute coronary occlusion is a major cause of cardiac arrest with ventricular fibrillation/ventricular tachycardia. However, this study separately analyzed OHCA patients with non-shockable and shockable rhythm and then, assessed the association between CAG and clinical outcomes in each rhythm group.

Recent study has reported that an initial shockable rhythm was the strongest indicator of acute coronary occlusion requiring early PCI [19]. CAG enables early identification and revascularization of coronary arterial occlusion, which lead to improve clinical outcomes in shockable OHCA [5]. In this study, PCI was performed in nearly 40% of patients who underwent CAG irrespective of initial rhythm. Performing CAG may have a similarly beneficial effect on clinical outcomes in non-shockable OHCA.

Routine CAG has widely applied in OHCA patients with ST elevation on the post-ROSC electrocardiogram (ECG) because an acute coronary occlusion was found in more than half of these patients [20] and an early coronary revascularization improved survival and neurological outcome in these patients who underwent CAG with PCI [21]. However, several studies showed a high incidence of significant coronary occlusion in OHCA patients without ST elevation on post-ROSC ECG, ranging from 26% to 58% and an improved survival in these patients who underwent CAG with PCI [18, 19, 22, 23]. Thus, it is difficult to assess an acute coronary occlusion as the cause of arrest using post-ROSC ECG in OHCA setting because it lacks sensitivity and specificity to predict an acute coronary occlusion [22–24]. According to the 2015 American Heart Association guidelines, CAG is recommend for all patients with ST elevation and for unstable patients without ST elevation on post-ROSC ECG after OHCA of suspected cardiac cause [25]. The 2014 European Society of Cardiology guidelines on myocardial revascularization have also recommended a routine immediate CAG in all OHCA survivors without an evident non-coronary cause irrespective of the post-ROSC ECG pattern [26]. One recent large cohort study emphasized the use of routine CAG in OHCA survivors as a standard post-cardiac arrest protocol [19]. Taken together, the present results may support the use of CAG for all OHCA survivors with suspected cardiac cause irrespective of initial cardiac rhythm. Further analysis may be needed to evaluate whether early CAG with revascularization improves clinical outcomes in non-shockable OHCA.

### Limitations

The present study has the following limitations. First, the study population size was relatively modest and especially the size of CAG group in non-shockable OHCA was small though this study used a nationwide multicenter registry. Second, post-ROSC ECG was missing in about half of CAPTURES registry. The indication for CAG were not pre-specified. Thus, it was possible that patients with STE on post-ROSC ECG were mainly selected for CAG by clinical assessment of physicians in each institution. It might lead to indication bias. Even PSM and IPTW analyses may be insufficient to count multiple factors such as functional status, comorbidities and family or social factors. Interestingly, INCAR registry, which was conducted in 34 centers in Europe and USA, showed 25% CAG rate for initial non-shockable rhythm, which is similar to 26% in our study [1]. However, to overcome selection or indication bias, further investigation whether early CAG with revascularization improves clinical outcomes in non-shockable OHCA would be required. Third, we used first cardiac rhythm documented by EMS, which did not account for patients’ arrest rhythm and subsequent rhythm changes between EMS arrival and hospital arrival. In this study, CAG group in non-shockable OHCA had more PEA and prehospital defibrillation, which might indicate that they had more changes to subsequent shockable rhythm before hospital arrival. In previous studies, OHCA with initial PEA and the change from non-shockable to shockable rhythm had better clinical outcome than OHCA with initial asystole and without the change from non-shockable to shockable rhythm, respectively [27, 28]. Thus, higher proportion of PEA and prehospital defibrillation might contribute to improved survival of CAG group in non-shockable OHCA. Nevertheless, to the best of our knowledge, no previous studies analyzed separately non-shockable and shockable rhythm to evaluate the association CAG and survival with good neurological outcome after OHCA. Therefore, our finding is notably significant due to the potential impact of CAG on treatment for OHCA survivors.

## Conclusion

In a Korean nationwide multicenter study of OHCA (CAPTURES), CAG was significantly associated with improved survival with good neurological outcome for adult OHCA patients of presumed cardiac cause with initial non-shockable rhythms. It suggests that CAG may be beneficial to adult non-shockable OHCA as well as to shockable OHCA. Further randomized controlled trials would be required to confirm the potential benefit of CAG and early revascularization for adult non-shockable OHCA.
